# Neonatal Erythroderma: Diagnostic Challenges and the Limitations of Genetic Testing

**DOI:** 10.7759/cureus.96134

**Published:** 2025-11-05

**Authors:** Jonathan Phipps, Otilia Popescu

**Affiliations:** 1 Neonatology, Sunderland Royal Hospital, Sunderland, GBR

**Keywords:** congenital ichthyosis, ichthyosis, ichthyosis care, icthyosiform erythroderma, newborn erythema

## Abstract

Neonatal erythroderma (NE) is an uncommon but serious presentation characterised by generalised erythema and scaling from birth. Its aetiology is diverse, encompassing congenital ichthyoses, infections, metabolic disorders, immunodeficiencies, and syndromic causes. We report the case of a full-term male neonate presenting shortly after birth with diffuse erythema, fine scaling, and areas of skin peeling, without mucosal involvement or systemic instability. Investigations, including biochemical studies, infection screening, and a next-generation sequencing ichthyosis panel, were unremarkable. Dermatology review favoured a diagnosis of congenital ichthyosiform erythroderma (CIE), and supportive management was initiated with regular emollients. The infant remained stable and was discharged with close multidisciplinary follow-up. Although the presentation was consistent with CIE, the clinical diagnosis remained uncertain at this stage. This case illustrates the diagnostic uncertainty inherent in NE and highlights the limitations of genetic testing in the neonatal period. Safe management centres on a structured multidisciplinary approach, longitudinal clinical assessment, parental education, and ongoing research to guide prognosis and therapy.

## Introduction

Neonatal erythroderma (NE) is a challenging presentation, characterised by widespread redness and scaling of the skin from birth. Rather than representing a single condition, it encompasses a broad differential diagnosis, including congenital ichthyoses, severe eczema, infections, metabolic disorders, immunodeficiencies, and syndromic causes [1-3]. For clinicians, the main challenge is to distinguish among these possibilities in the neonatal period, when clinical signs are often non-specific and genetic results are delayed [4]. Establishing the aetiology can be difficult due to the poor specificity of clinical and histological signs, and the time to diagnosis is often prolonged, with reports citing delays of up to 116 days in cases of genetic erythroderma [4,5]. Early and accurate diagnosis is crucial, as affected infants are vulnerable to electrolyte disturbances, infection, and dehydration due to impaired skin-barrier function, with much greater transepidermal water loss than that of a normal full-term infant [6]. While advances in genetic testing have transformed the evaluation of NE, reliance on next-generation sequencing panels alone has limitations: turnaround times may be prolonged, and 10-15% of clinically typical cases remain genetically unexplained [7,8]. Structured diagnostic frameworks can provide a clinically useful algorithm to guide initial evaluation and management while awaiting genetic confirmation [4].

Among inherited causes, congenital ichthyosiform erythroderma (CIE) remains an important consideration. CIE belongs to the autosomal recessive congenital ichthyoses (ARCIs), a rare group of disorders characterised by abnormal cornification and impaired barrier function [7]. Affected infants usually show widespread erythema and scaling at birth. A collodion membrane is a recognised feature of ARCI, historically reported in more than 90% of cases of CIE [9]. However, more recent data suggest a prevalence of approximately 53% in the CIE subgroup [7]. Several causative genes have been identified, including *TGM1*, *ALOX12B*, *ALOXE3*, and *ABCA12*, though a subset of cases remains genetically unexplained [7,8].

While management of NE is mainly supportive, with a focus on maintaining hydration, reducing scaling, and preventing infection, the accuracy of diagnosis has important implications for prognosis, parental counselling, and long-term care [10]. This case highlights the diagnostic uncertainty inherent in NE, particularly when clinical features overlap and genetic confirmation is unavailable, emphasising the importance of careful clinical assessment and a multidisciplinary approach.

## Case presentation

A baby boy born at term via spontaneous vaginal delivery was reviewed at four hours of age, after widespread erythema, blistering, and peeling were noted. He was born in good condition and required no medical intervention at delivery. Apgar scores were 9 at one and 1 at ten minutes of age; he lost only one point from the appearance assessment, of the available two, for normal physiological peripheral acrocyanosis. Birth weight and length were recorded as 3.37 kg and 52 cm, respectively, both plotting >50th centile for gestation. Initial observations recorded oxygen saturation of 100% in air, heart rate of 112 beats/minute, respiratory rate of 45 breaths/minute, and temperature of 36.6°C. This was plotted on the neonatal early warning score (NEWS) tool with a rating of 0, indicating low clinical concern. The child was reported to be breastfeeding well and was not irritable or showing evidence of discomfort or pain; therefore, further objective pain assessment tools were not used. No additional concerns were raised by the parents or the midwifery team.

There was no significant antenatal history; this was a low- to moderate-risk, midwife-led pregnancy, due to a previous child born small for gestational age, and serial growth scans were undertaken, all normal. The mother did not receive medications other than routine folic acid and multivitamin supplements; there was no history of consanguinity. The only family history of note was eczema in the baby’s sibling.

**Figure 1:**
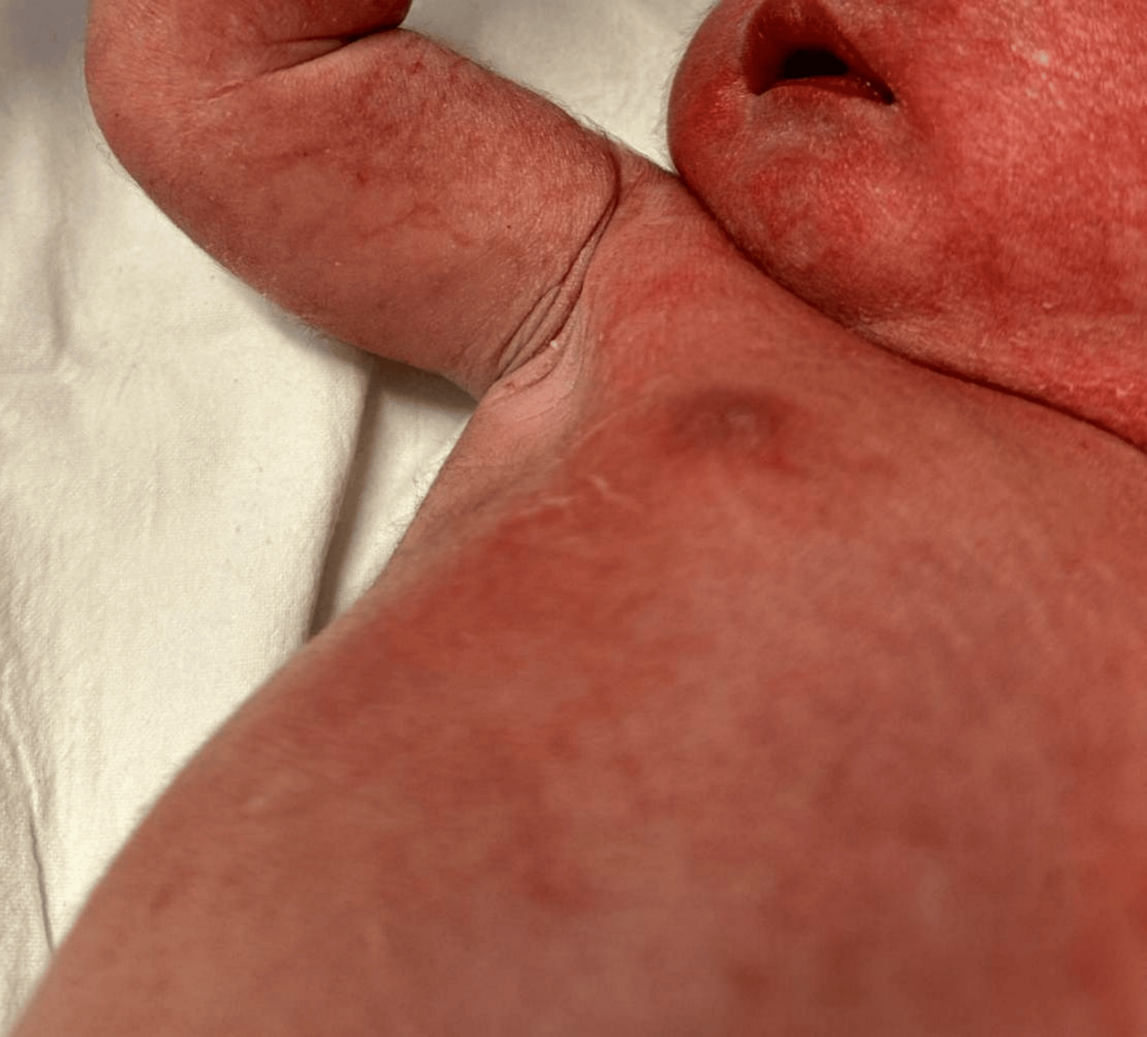
General texture of the child’s skin with widespread erythema and scaling.

**Figure 2:**
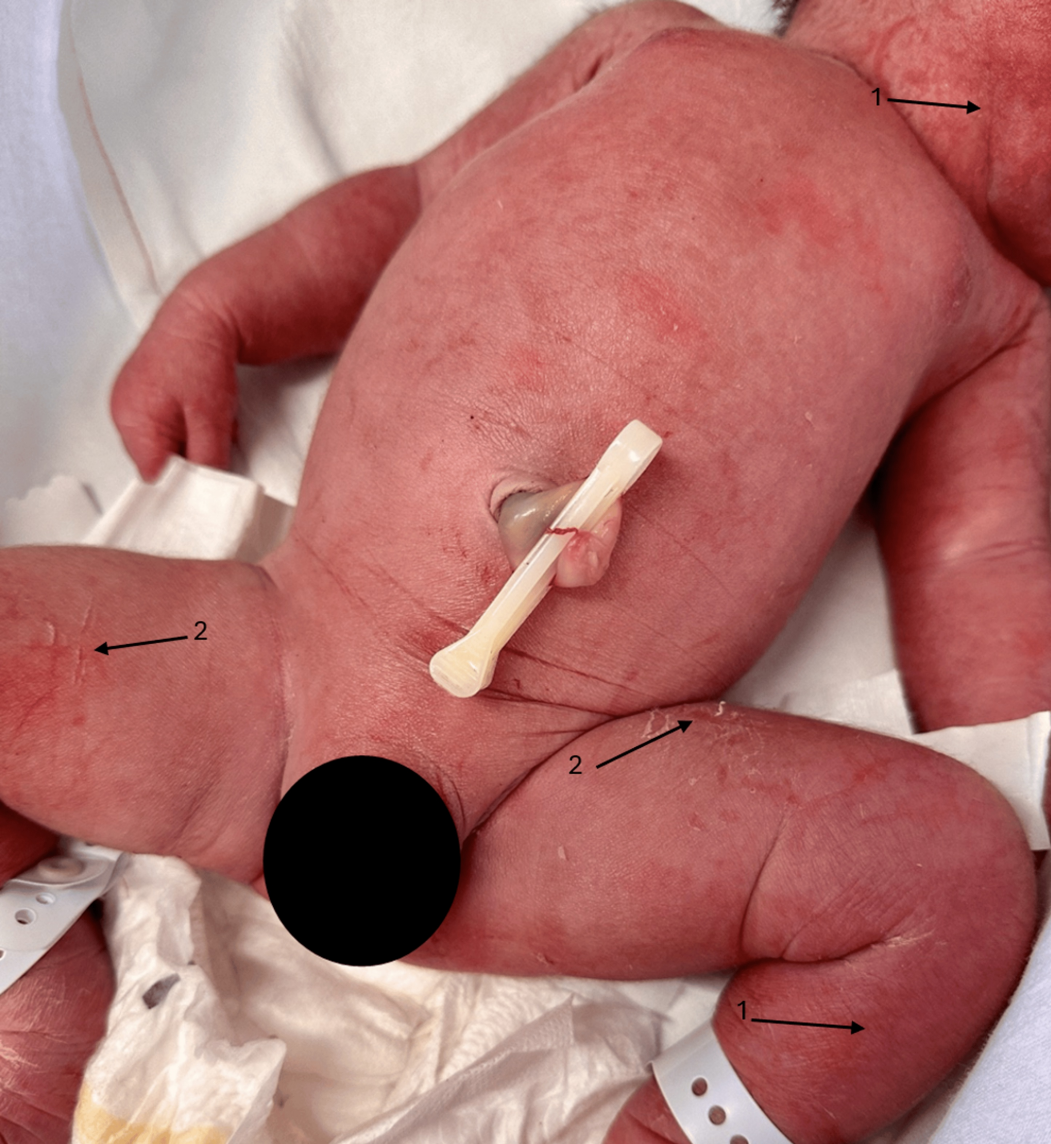
Widespread erythema and erosions on the legs. Arrow 1: areas or increased erythema. Arrow 2: areas of superficial erosion.

**Figure 3:**
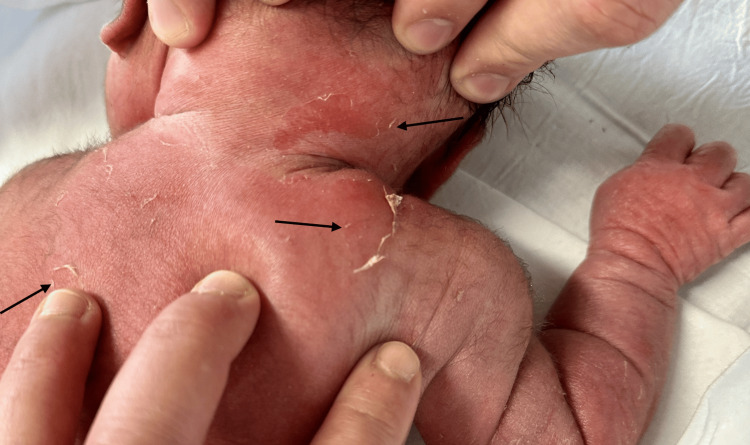
Areas of blistering and erosion on the back and neck.

On initial examination by the neonatal clinician, the child was reported to have general erythema covering the entire surface of the skin, with a shiny, scaly texture. The consistency was generally soft but reported to be firmer in the more erythematous areas, such as on the face and lower limbs, as seen in Figure 1 and Figure 2. Blistering of the skin was noted around the forehead and neck with areas of epidermal erosion predominantly on the back, lower limbs, and scrotum, as shown in Figure 2 and Figure 3. There was no evidence of tenderness or discomfort on palpation, no friction-related erosion (negative Nikolsky sign), and no mucosal involvement. The skin was of a normal temperature throughout, including the areas of more pronounced erythema, and there was no evidence of oedema or discharge. Physiological parameters remained normal as per the NEWS tool chart, and general examination revealed no features to suggest systemic infection or any life-threatening condition.

The neonatal team continued to monitor the baby, and dermatology input was sought. Blood tests, including capillary blood gas, full blood count, urea and electrolytes, liver function tests, bone profile, total immunoglobulin E (IgE), and zinc levels, were performed. Initial capillary blood gas results were reassuring, with normal pH, CO₂, and O₂ levels. Glucose and lactate were within expected ranges. There was erythrocytosis with elevated haematocrit; hydration was monitored, and good feeding and urine output were observed. Venous haematocrit was reassuringly normal, not indicating a true polycythaemia. Other pertinent results were an initially raised potassium, felt by the biochemist to be due to partial haemolysis of the blood sample, and a slightly raised chloride, which was normal on subsequent capillary blood gas analysis. All pertinent results are listed in Table 1; appropriate neonatal reference ranges are provided as per the local laboratory.

**Table 1:** Initial and significant laboratory findings with normal reference ranges.

Capillary blood gas	Value	Reference range	Venous blood test	Value	Reference range	Test	Result
Capillary blood pH	7.4	7.35–7.45	White blood cell count	9.69 × 10⁹/L	4.0–11.0 × 10⁹/L	Bacterology and fungi	Moderate growth of skin flora
Capillary blood pCO₂	6.0 kPa	4.7–6.0 kPa	Red blood cell count	6.47 × 10⁹/L	4.0–6.6 × 10⁹/L	Enterococcus PCR	Not detected
Capillary blood PO₂	8.1 kPa	8–10.6 kPa	Platelet count	202 × 10⁹/L	150–450 × 10⁹/L	Syphilis PCR	Not detected
Capillary blood glucose	3.4 mmol/L	2.0–6.0 mmol/L	Mean corpuscular volume	97.5 fL	95–121 fL	Herpes simplex type 1 PCR	Not detected
Capillary blood sodium	141 mmol/L	133–146 mmol/L	Haemoglobin	239 g/L	145–225 g/L	Herpes simplex type 2 PCR	Not detected
Capillary blood haemoglobin	252.0 g/L	15–178 g/L	Haematocrit	0.634 L/L	0.45–0.67 L/L	Varicella Zoster PCR	Not detected
Capillary blood haematocrit	72%	36–53 %	Absolute eosinophils	0.29 × 10⁹/L	0.1–2.5 × 10⁹/L		
Capillary lactate	1.9 mmol/L	0.2–2.0 mmol/L	Alkaline phosphatase	225 U/L	83–248 U/L		
Capillary blood bicarbonate	28.0 mmol/L	22–32 mmol/L	Total bilirubin	276 µmol/L	0–200 µmol/L		
Capillary potassium	5.20 mmol/L	3.4–4.5 mmol/L	Sodium level	141 mmol/L	133–146 mmol/L		
			Potassium level	4.2 mmol/L	3.4–4.5 mmol/L		
			Chloride level	109 mmol/L	95–108 mmol/L		
			Creatinine	35 µmol/L	27–90 µmol/L		
			Urea level	1.9 mmol/L	0.8–5.5 mmol/L		
			Plasma zinc level	9.0 µmol/L	5–21.5 µmol/L		
			Total IgE antibody	0.2 IU/ml	0–10.9 IU/ml		

As an inpatient, there was minimal change in the child’s skin condition; however, he remained under daily review by the neonatal team with capillary blood gas sampling to monitor for electrolyte abnormalities. He continued to breastfeed well, showing no irritability or discomfort, and all observations remained within normal parameters as per the NEWS tool. On day five of life, he was discharged home with a plan for frequent follow-up with the dermatology team and the neonatal consultant.

He was reviewed by dermatology soon after discharge, and a next-generation gene sequencing, R165 ichthyosis and erythrokeratoderma genetics panel was completed, assessing 54 common genes associated with the disease. There was no evidence of known mutations in these genes (Table 2).

**Table 2:** Genes analysed as a part of the R165 ichthyosis and erythrokeratoderma genetics panel.

Gene	Gene	Gene	Gene	Gene	Gene
AAGAB	CERS3	FLG2	KRT1	LORICRIN	SLURP1
ABCA12	CLDN1	GJA1	KRT10	NIPAL4	SNAP29
ALDH3A2	CYP4F22	GJB2	KRT14	PIGL	SPINK5
ALOX12B	DSC2	GJB3	KRT16	PNPLA1	ST14
ALOXE3	DSG1	GJB4	KRT17	RHBDF2	STS
AQPS	DSP	GJB6	KRT6A	RSPO1	SULT2B1
ASPRV1	ELOVL1	GTF2E2	KRT6B	SDR9C7	TAT
CARD14	ENPP1	JUP	KRT6C	SERPINB7	TGM1
CAST	FLG	KDSR	KRT9	SLC27A4	TRPV3

Further management with steroids or retinoids was deferred at this point due to parental concerns of potential side effects, with a plan to continue discussions with dermatology and review if the patient’s condition worsened.

A collection of hair was cut from the child’s scalp and examined under a light microscope. This showed occasional foci of partial shaft fracture and fraying, with early trichorrhexis nodosa lesions. This was felt to be a non-specific finding of fragility and is known to be consistent with many causes of NE, including ichthyosis, Netherton syndrome (NS), trichothiodystrophy (TTD), and other genetic causes [4,11]. There was no evidence of banding seen under polarised light, indicative of TTD or trichorrhexis invaginata, bamboo hair, which would have been suggestive of NS [4]. The day five blood spot screen was performed and showed no evidence of the 10 rare conditions evaluated. Invasive skin biopsy and immunofluorescence have at this point not been consented for and the diagnosis remains uncertain.

The child remains under tight monthly follow-up with dermatology, and treatment has been slowly escalated in view of ongoing erythema despite appropriate management. His current management includes topical 1% hydrocortisone for erythema, topical 0.05% clobetasone butyrate for areas of pronounced scaling, pimecrolimus cream as an immunomodulator to manage erythema, regular application of a paraffin-based emollient, and aqueous cream for bathing. He remains under close follow-up with the neonatal consultant, and parents have been given contact information for the local paediatrics team and are encouraged to contact them if they have any concerns.

Management is intensive and requires significant commitment from the parents; however, they are satisfied to see gradual clinical improvement in his symptoms.

## Discussion

One of the main challenges in this case was correlating the clinical picture with a specific diagnosis. A clear diagnosis guides management, improves outcomes, and provides reassurance to families [12]. While a preliminary diagnosis of CIE was made, it remained uncertain after extensive investigation. The patient was, however, managed safely and appropriately throughout admission based on observed signs and available results.

NE is defined as erythema and generalised dermatitis affecting at least 90% of the body surface area at birth or within the first four weeks of life [13]. Its differential includes several potentially life-threatening conditions, making a multidisciplinary approach essential with involvement of paediatricians and dermatologists, as undertaken in this case [4]. Infection was appropriately ruled out through skin swabs and careful monitoring. The infant remained stable, with no evidence of dehydration or electrolyte imbalance; this allowed safe management in transitional care. Emollient therapy was commenced, and neonatal intensive care unit admission was not required, allowing the baby to remain with his parents and establish breastfeeding successfully.

Diagnostic uncertainty and the role of genetic testing

The differential diagnosis for NE includes other ARCI variants such as lamellar and harlequin ichthyosis, as well as NS, severe atopic dermatitis, and psoriatic erythroderma. Non-genetic causes, including infections, metabolic disorders, and immunodeficiencies, also warrant consideration [2]. A diagnosis can be difficult to establish, and even with advances in genomic analysis, the time to diagnosis may take months, with the longest delays seen in non-syndromic ichthyoses [4].

In this case, the infant’s systemic stability, laboratory results within expected limits, a negative newborn blood spot screen, and negative infection screens argued against syndromic or metabolic causes. Normal IgE levels and absence of characteristic “bamboo hair” shaft anomalies made NS less likely [14]. The absence of a collodion membrane added diagnostic complexity; however, it does not exclude CIE.

CIE is part of the ARCI spectrum, a genetically heterogeneous group of disorders caused by mutations in over 20 genes affecting lipid metabolism and cornified envelope formation [2,7]. The negative R165 ichthyosis and erythrokeratoderma panel does not rule out the condition, as targeted panels may miss deep intronic, structural, or previously unidentified variants [7,15]. Approximately 10-15% of clinically typical cases remain genetically unresolved [4,7].

Broader genomic methods, such as whole-exome and whole-genome sequencing, can identify novel variants [4,8]. However, these techniques are costly, require sophisticated analysis, and have long turnaround times, limiting their usefulness in the acute neonatal setting. Consequently, early management is often based on the clinical phenotype, with ongoing monitoring and tertiary dermatology input [4].

Management and therapeutic rationale

Without a definitive diagnosis, treatment was symptomatic and reactive. Management of all causes of NE focuses on maintaining hydration, supporting the skin barrier, and preventing infection [3,16]. Regular application of a bland paraffin-based emollient four times daily reduces xerosis and improves comfort. Emollients remain the cornerstone of care in both NE and ARCI, reducing scaling and transepidermal water loss while improving skin flexibility [11,16].

Systemic retinoids such as acitretin were not initiated, as there was appropriate parental concern about the potential side effects and toxicity, which need to be weighed against the current symptoms and absence of a confirmed diagnosis [8,16,17]. Keratolytic preparations containing urea or salicylic acid were avoided due to risks of irritation and systemic absorption with a compromised epidermis [8,17]. Conservative management was appropriate at this stage; given the infant’s stable condition and plan for extensive follow-up, we were able to monitor for improvement and introduce additional treatment if necessary. Keratolytics and retinoids may need to be considered if hyperkeratosis were to become a more predominant feature, especially if not responsive to safer topical treatments.

Phenotypic considerations

Phenotypic variability among congenital ichthyoses significantly influences early management. Infants with more severe phenotypes, particularly those with collodion membranes, require close monitoring in a humidified incubator to minimise transepidermal water loss and temperature instability, and to detect infection or electrolyte disturbances promptly [1,17]. Multidisciplinary collaboration between dermatology, neonatology, and genetics remains key to achieving optimal outcomes.

Consequences of diagnostic ambiguity

A genetically unresolved diagnosis presents both practical and emotional challenges. Without confirmation, prognosis and recurrence risk remain uncertain, which can cause anxiety for families regarding future pregnancies. Effective communication and close follow-up are essential, ensuring parents understand the provisional nature of the diagnosis and the potential for future genomic results to clarify it [10]. Periodic re-analysis or broader genomic testing may ultimately provide diagnostic certainty. Meanwhile, longitudinal clinical assessment remains the most reliable method to monitor disease progression and guide care. As a significant improvement has not yet been observed, monthly follow-up continues to monitor stability and determine when less frequent reviews may be appropriate.

The literature reports multiple instances of successful management of suspected ichthyosis without genetic confirmation [18,19]. They highlight the need for management that is responsive to clinical findings and illustrate the necessity of careful monitoring, especially in the initial neonatal period.

## Conclusions

This case illustrates the ongoing diagnostic and management challenges associated with NE. Given the wide differential diagnosis, overlapping clinical features, and limited availability of rapid genetic testing, establishing a definitive diagnosis in the neonatal period remains challenging. Accordingly, close and repeated clinical assessment is essential; management should be guided by the evolving clinical picture while awaiting diagnostic confirmation. Infants should be carefully monitored for life-threatening infections and electrolyte disturbances, with a multidisciplinary approach involving dermatology, neonatology, midwifery, and genetics services. Despite features strongly suggestive of CIE, the absence of a collodion membrane and a negative genetic panel at present leaves the diagnosis uncertain. The child will continue to be under follow-up, and broader sequencing approaches or skin biopsy with histopathology may ultimately define the molecular defect in this case. Until then, management must remain phenotype-driven and supportive. Regular emollient therapy, multidisciplinary follow-up, and sensitive parental education remain central to achieving optimal outcomes.
